# Human immunodeficiency virus Tat associates with a specific set of cellular RNAs

**DOI:** 10.1186/1742-4690-11-53

**Published:** 2014-07-03

**Authors:** Russell D Bouwman, Anne Palser, Chris M Parry, Eve Coulter, Jane Rasaiyaah, Paul Kellam, Richard G Jenner

**Affiliations:** 1 MRC Centre for Medical Molecular Virology, Division of Infection and Immunity, University College London, London WC1E 6BT, UK; 2 Wellcome Trust Sanger Institute, Hinxton, Cambridge CB10 1SA, UK; 3 Health Protection Agency, 61 Colindale Avenue, London NW9 5EQ, UK; 4 Current Address: MRC/UVRI Uganda Research Unit on AIDS, Uganda Virus Research Institute, Entebbe, Uganda; 5 UCL Cancer Institute, University College London, London WC1E 6BT, UK

**Keywords:** Human immunodeficiency virus, Tat, RNA, Transcription factor, RNA immunoprecipitation, Microarray, T cell, MicroRNA

## Abstract

**Background:**

Human Immunodeficiency Virus 1 (HIV-1) exhibits a wide range of interactions with the host cell but whether viral proteins interact with cellular RNA is not clear. A candidate interacting factor is the trans-activator of transcription (Tat) protein. Tat is required for expression of virus genes but activates transcription through an unusual mechanism; binding to an RNA stem-loop, the transactivation response element (TAR), with the host elongation factor P-TEFb. HIV-1 Tat has also been shown to alter the expression of host genes during infection, contributing to viral pathogenesis but, whether Tat also interacts with cellular RNAs is unknown.

**Results:**

Using RNA immunoprecipitation coupled with microarray analysis, we have discovered that HIV-1 Tat is associated with a specific set of human mRNAs in T cells. mRNAs bound by Tat share a stem-loop structural element and encode proteins with common biological roles. In contrast, we do not find evidence that Tat associates with microRNAs or the RNA-induced silencing complex (RISC). The interaction of Tat with cellular RNA requires an intact RNA binding domain and Tat RNA binding is linked to an increase in RNA abundance in cell lines and during infection of primary CD4+ T cells by HIV.

**Conclusions:**

We conclude that Tat interacts with a specific set of human mRNAs in T cells, many of which show changes in abundance in response to Tat and HIV infection. This work uncovers a previously unrecognised interaction between HIV and its host that may contribute to viral alteration of the host cellular environment.

## Background

Like all viruses, HIV-1 depends on the internal environment of the host cell to complete its life cycle and during virus replication a number of interactions occur between the virus and its host. These include interactions between viral and cellular proteins and viral proteins and host DNA. Whether HIV encodes proteins that allow interaction with cellular mRNA is unknown.

HIV Tat activates transcription of viral messenger RNA (mRNA) and genomic RNA from the integrated provirus and is necessary for HIV replication [1,2]. In the absence of Tat, RNA polymerase II initiates transcription at the HIV long terminal repeat (LTR) but processive elongation is blocked, leading to the production of short RNA transcripts [3,4]. This stalled transcriptional state also occurs in latently infected resting CD4+ T cells [4,5] and is thought to underlie life-long virus persistence.

Tat localizes to the LTR through an unusual mechanism, binding to the trans-activation response element (TAR), an RNA stem loop formed in nascent viral transcripts [6-8]. Tat binds to TAR with the host elongation factor P-TEFb through direct interaction with the CyclinT1 subunit [9-12]. P-TEFb then phosphorylates the RNA polymerase II C-terminal domain and the repressive factors NELF and DSIF, allowing transcriptional elongation. Tat also directly binds and recruits histone acetyltransferases [13] and the nucleosome remodeling complex SWI/SNF [14,15]. These changes in chromatin conformation allow full transcription of HIV messenger RNA (mRNA) and genomic RNA and subsequent release of infectious virions.

HIV Tat has also been suggested to function as a suppressor of post-transcriptional gene silencing (PTGS) [16-19], interacting with Dicer [17], but these findings have not been confirmed by other studies [20,21]. Such activity may counter reported repression of HIV replication by the cellular PTGS pathway [16-19,22-27]. HIV RNA has been observed to be recruited to cellular mRNA processing bodies (P-bodies) [22] and knockdown of RNA induced silencing complex (RISC) and P-body components [21-24], or inhibition of specific cellular miRNAs [25-27], have been described to increase HIV RNA abundance and viral replication. However, a more recent study did not observe co-localisation of HIV RNA with P-bodies or observe effects of P-body component knockdown on HIV replication [28].

In addition to binding to TAR, Tat has the potential to interact with host RNAs but whether this occurs in cells is unknown. Although the high affinity of Tat for TAR requires the stem-loop structure and a bulge of unpaired nucleotides present on the 5’ side of the loop, Tat can tolerate a range of mutations to the TAR sequence [29,30] and this may allow interaction with host RNA to occur in cells. Consistent with this possibility, Tat has been reported to bind to 3 cellular mRNA molecules, all *in vitro.* Recombinant Tat binds to a predicted stem loop at the 5’ end of IL6 mRNA *in vitro* and this sequence is necessary for Tat-mediated increase in IL6 RNA *in vivo*[31]. Similarly, recombinant Tat binds TLR4 RNA *in vitro*[32], Tat-containing cell lysate shifts a SOD2 RNA probe [33] and Tat-mediated activation of *LTA* (*TNFβ*) requires the presence of a predicted stem-loop structure at the 5’ end of its mRNA [34].

Expression of HIV Tat in cells leads to changes in cellular gene expression that often mirror the changes that occur during HIV infection [35-39]. Given that Tat has also been reported to interact with a wide range of cellular proteins, including components of the basal transcription machinery [40,41], transcription factors [42,43], chromatin regulators [44,45], anti-viral factors [46,47], and cell surface receptors [48,49], it is unclear whether effects of Tat on the levels of cellular mRNAs reflect direct Tat-RNA interactions.

Considering that Tat is an RNA binding protein and that it can alter cellular RNA abundance, we hypothesized that Tat binding to human RNA might represent an uncharacterized interaction between HIV and its host. We report here experiments revealing that HIV Tat associates with a specific set of cellular mRNAs in T cells.

## Results

### Tat associates with human mRNA in T cells

**Figure 1:**
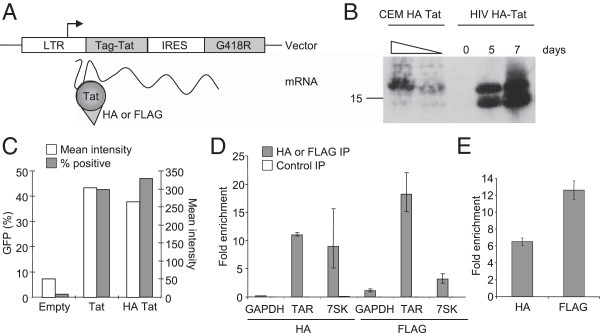
**A system for identifying human RNAs bound by HIV Tat. A**. HA or FLAG-tagged Tat was stably expressed from the HIV LTR as part of a bicistronic transcript with a G418 resistance gene in CEM T cells. Tat protein bound to the TAR stem-loop present at the 5’ end of the transcript. **B**. Western blotting for HA-Tat in CEM HA-Tat cells (1/1 and 1/5 dilutions) and in an equal number of C1866 cells 0, 5 and 7 days after infection with the SF2 strain of HIV engineered to encode HA-tagged Tat. **C**. Activity of untagged and HA-tagged Tat measured by GFP expression from the HIV LTR in the GHOST reporter line using flow cytometry. **D**. Enrichment (mean and SD) of TAR-Tat construct, 7SK and GAPDH RNA in HA-Tat and FLAG-Tat IPs compared to input RNA measured by Q-PCR. 7SK is enriched by 9-fold (SD 3.8) by IP for HA-tagged Tat and 3.6-fold (SD 0.73) by FLAG-tagged Tat. In comparison, GAPDH RNA is enriched by 0.2-fold (SD 0.007) and 1.14-fold (SD 0.25), respectively. Enrichment of 7SK compared to GAPDH is significant for both HA and FLAG IPs (p = 0.017 and 0.0051, respectively, t-test, n = 3). The enrichment in control IPs where the antibodies were switched are shown for comparison (white bars). **E**. Enrichment (mean and SD) of poly-adenylated TAR-Tat construct RNA in HA-Tat and FLAG-Tat IPs relative to input RNA measured using microarrays.

We hypothesized that Tat interacts with human RNA in HIV-1 target cells. To test this, we generated human T cell lines in which HA or FLAG epitope-tagged Tat is stably expressed under the control of the HIV-1 LTR (Figure 1A). Expression from the HIV-1 LTR ensures physiological levels of Tat expression (Figure 1B) and epitope-tagging does not alter protein activity (Figure 1C). Tat was then immunoprecipitated (IP) with anti-HA or anti-FLAG antibodies and bound RNAs purified. Enrichment of TAR, encoded by the HIV-1 LTR, in the IP could be measured by quantitative polymerase chain reaction (Q-PCR) and acted as a positive control for the assay (Figure 1D). IP from HA-Tat cells with anti-FLAG antibody, or from FLAG-Tat cells with anti-HA antibody, did not result in significant RNA purification, demonstrating that both antibodies specifically enrich for Tat. We could also detect enrichment of cellular 7SK RNA (Figure 1D), with which Tat associates through P-TEFb [50].

**Figure 2:**
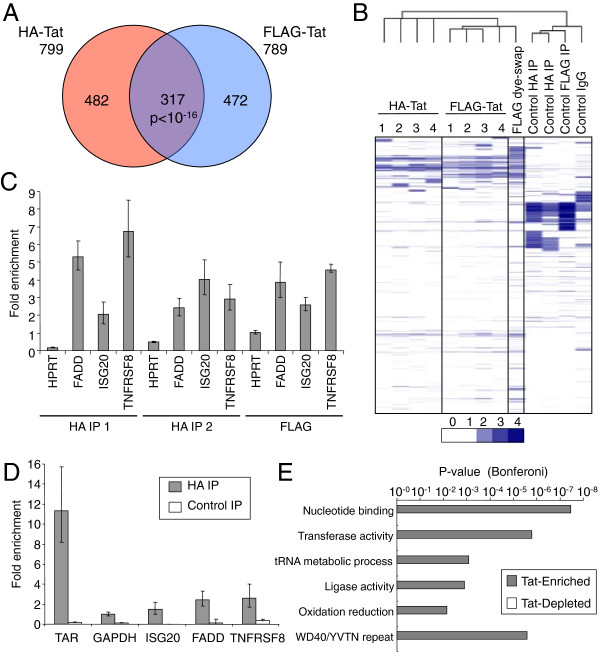
**Tat interacts with human mRNA in cells. A**. Venn diagram showing cellular RefSeq mRNAs enriched by IP for HA-Tat or FLAG-Tat. The overlap between the two sets of RNAs is significant at p < 10^-16^ (Hypergeometric). **B**. Clustered heat-map of the RNA IP data. Fold-enrichment of mRNA in the IP fraction relative to input RNA is shown as shades of blue, according to the scale below. Samples (columns) and mRNAs (rows) are clustered by their enrichment pattern with genes and samples with similar patterns of enrichment grouped together. The numbers 1–4 signify the 4 different probes present on the arrays used to measure enrichment by HA and FLAG-Tat IP. The arrays used for the other experiments only contain probe number 4. The similarities between the pattern of RNA enrichment for each experiment is related by the tree shown above. **C**. Enrichment (mean and SD) of the human mRNAs FADD, ISG20 and TNFRSF8 in replicate HA-Tat and FLAG-Tat IPs relative to GAPDH mRNA, measured by Q-PCR. An additional negative control, HPRT mRNA, was enriched between 0.2-1-fold. **D**. Enrichment (mean and SD) of the human mRNAs GAPDH, FADD, ISG20 and TNFRSF8 in HA-Tat immunoprecipitate from formaldehyde cross-linked cells, measured by Q-PCR. The p-values for enrichment versus GAPDH were TAR RNA p = 0.0046, ISG20 p = 0.1778, FADD p = 0.0185 and TNFRSF8 p = 0.0395 (t-test, n = 3). Enrichment of RNA by anti-HA IP from FLAG-Tat cells was performed as a negative control. **E**. Bonferoni-modified p-value for the association of functional gene categories with enlarged sets of ~2000 genes enriched or depleted in HA-Tat and FLAG-Tat IPs. .

To identify human mRNA associated with Tat, we differentially labelled IP-enriched mRNA and total cellular mRNA and hybridized these together to a microarray containing multiple probes for all human RefSeq transcripts. The array also contained probes for HIV RNA, which confirmed enrichment of TAR by Tat IP (Figure 1E). Because only poly-adenylated RNAs are labelled for the array analysis (due to the use of oligo-dT to prime cDNA synthesis), this detection of Tat binding to TAR demonstrates that Tat remains associated with HIV RNA after poly-adenylation has taken place. We then analysed the data from the human RNA probes. Comparison of HA Tat and FLAG Tat RNA IPs revealed a significant overlap between the cellular RNAs enriched in both experiments (Figure 2A). Requiring at least two probes to report significant enrichment in the IP RNA fraction, we identified a total of 317 cellular mRNAs associated with Tat in both HA and FLAG IPs (of 16,538 mRNAs present on the array, Additional file 1). Therefore, in addition to binding to TAR, Tat also interacts with host RNAs. Furthermore, rather than binding to all mRNAs, Tat binds to a specific set of transcripts. This set of RNAs bound by Tat included those encoding for ISG20, JUN, DNAJB2, FADD, TNFRSF8 and OAS3, expression of which have previously been shown to be regulated by Tat [35-39]. The inclusion of genes previously found to be regulated by Tat provides confidence in our data and suggests that Tat may mediate changes in RNA abundance through direct interaction with specific mRNA species.We swapped labelling dyes to ensure that the apparent enrichment of cellular RNA was not due to biases in dye incorporation; 83% of RefSeq mRNAs identified in the original Tat RNA IPs were also identified in this dye-swap experiment (Figure 2B). To further verify that Tat specifically interacted with these cellular RNAs, we labelled the RNA purified in our control IP experiments (anti-HA IP from FLAG-Tat cells, anti-FLAG IP from HA-Tat cells and control Rat IgG IP from HA-Tat cells). Unlike Tat-enriched RNA, the small amounts of RNA enriched by these control IP experiments did not produce material sufficient for hybridisation according to the standard protocol. However, to confirm that even this small amount of control IP material did not exhibit enrichment for Tat-associated RNAs, we hybridized the labelled RNA to the arrays. The use of cluster analysis to compare these data to our three Tat RNA IP experiments demonstrated that control IPs produce distinct patterns of enrichment that do not correlate with the RNAs enriched by Tat IP (Figure 2B).

We next sought to verify the array results by measuring enrichment of individual RNAs by Q-PCR (Figure 2C). We noted that RNAs encoding ISG20 and the apoptosis mediators TNFRSF8 (CD30) and FADD, previously shown to be upregulated by HIV Tat [36,39], were bound by Tat in both array experiments. QPCR for TNFRSF8, FADD and ISG20 mRNA in replicate HA and FLAG Tat IPs confirmed enrichment of these RNAs compared to GAPDH mRNA. Also consistent with the array data, qPCR for HPRT1 mRNA showed that this was not enriched compared to GAPDH mRNA (between 0.2-1-fold). The arrays therefore accurately report enrichment of specific mRNAs in Tat IP fractions.There is a potential with native RNA IP experiments that proteins form new interactions with RNA after cell lysis that did not occur in the intact living cell. To test whether Tat also interacted with human RNAs within intact cells, we repeated the RNA IP after crosslinking the cells with formaldehyde (Figure 2D). These experiments showed that FADD and TNFRSF8 remain enriched in IPs from crosslinked cells and therefore that Tat interacts with these RNAs in living cells.

### Tat-associated human RNAs are enriched for specific functions

We next used Gene Ontology to explore whether the set of RNAs that interact with Tat had any functions in common (Figure 2E). To provide robust statistics for this assessment, we used cluster analysis to identify a larger set of 2000 RNAs enriched in both HA and FLAG RNA IPs and a control set of 2000 RNAs depleted in HA and FLAG IPs. Gene categories specifically enriched in the set of Tat-associated RNAs included nucleotide binding and the overlapping categories transferase activity, tRNA metabolic process and ligase activity. Indeed, this set of RNAs included 26 of the 34 enzymes that constitute the KEGG aminoacyl-tRNA biosynthesis pathway (Additional file 2). The enrichment of mRNAs encoding shared functional annotations suggests that they may share specific sequence or structural features or a common RNA binding protein that mediates interaction with HIV Tat.

### Tat does not associate with miRNAs

**Figure 3:**
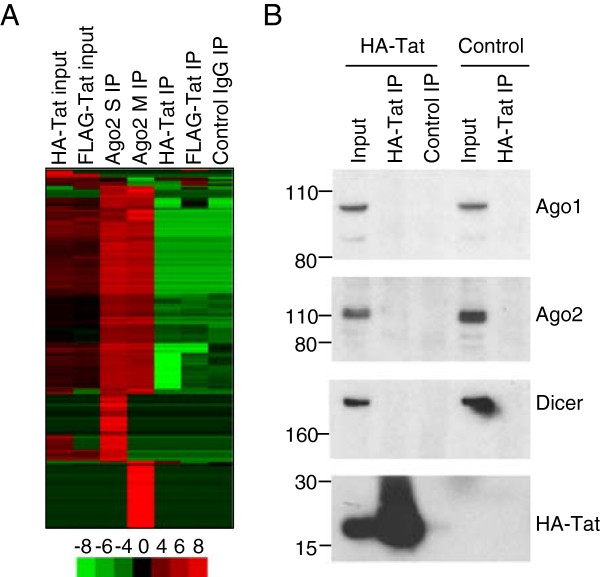
**Tat does not associate with miRNAs or with RISC components. A**. Clustered heat-map showing the amount of cellular miRNAs in HA-Tat and FLAG-Tat cells and in Ago2, Tat and control IgG IP. Ago2 was immunoprecipitated with two different antibodies (M and S). Samples (columns) and miRNAs (rows) are clustered by their enrichment pattern. miRNA abundance compared to the average across all samples is indicated by colour, according to the scale below (log2 ratio). **B**. Immunoblotting for Ago1, Ago2, Dicer and HA-Tat in input and HA-Tat IP lysates from HA-Tat cells and control FLAG-Tat cells. The position of size markers are illustrated on the left hand side of each panel.

The results described above show that HIV Tat associates with specific mRNAs. Tat has been reported to interact with exogenously expressed Dicer [17] and TAR is processed into small RNA species [51-54], suggesting that Tat might also interact with miRNAs, either directly or through RISC components. To test this, HA-Tat and FLAG-Tat were immunoprecipitated as before and the IP RNA labelled and hybridised to miRNA arrays, together with input and control IP RNA (Figure 3A). As a positive control, the RISC component Ago2 was precipitated from FLAG-Tat cells with two independent antibodies. We found that IP for Ago2 strongly enriched for miRNAs, an average of 15-20-fold. However, no miRNAs were enriched by IP for FLAG or HA-tagged Tat and the results were indistinguishable from the negative control IP. We conclude that, contrary to its interaction with host mRNA, Tat does not interact with host miRNA species.The lack of association between Tat and miRNAs suggested that Tat does not interact with RISC components. To confirm this, we immunoprecipitated HA Tat and tested for co-purifying endogenous Dicer, Ago1 and Ago2 by western blotting (Figure 3B). We could not detect any of these components of RISC co-precipitating with Tat and, taken together with the miRNA IP data, this indicates that Tat is not present in a ribonucleoprotein complex with RISC in T cells.

### The interaction of Tat with cellular RNA depends on its RNA binding domain

**Figure 4:**
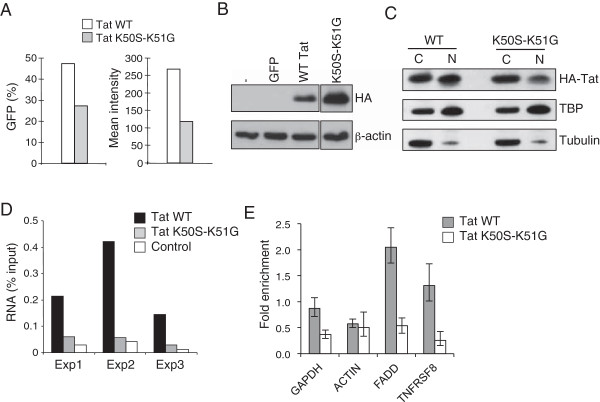
**The interaction of Tat with human RNA depends on the RNA binding domain. A**. Activity of wild-type HA-Tat (white bars) and HA-Tat K50S-K51G (RNA binding mutant), grey bars) in the LTR reporter cell line GHOST, measured by the proportion of GFP-positive cells (left panel) and mean GFP intensity (right panel). **B**. Immunoblotting for HA-Tat and β-actin in CEM cells 72 hours after infection with lentiviral vectors expressing either GFP alone, GFP and WT HA-Tat or GFP and HA-Tat-K50S-K51G. **C**. Immunoblotting for HA-Tat, TBP (nuclear protein) and Tubulin (cytoplasmic protein) in cytoplasmic (C) and nuclear (N) fractions of CEM cells expressing WT HA-Tat or HA-Tat-K50S-K51G. **D**. Amount of DNase-treated RNA purified from HA-Tat and control antibody IPs from CEM cells infected with WT-Tat or Tat K50S-K51G encoding lentivirus, as a percentage of the total RNA in the lysate. Data from 3 independent infections are shown. **E**. Enrichment (mean and SD) of the human mRNAs GAPDH, ACTIN, FADD and TNFRSF8 in HA-Tat immunoprecipitate from cells infected with WT HA-Tat or HA-Tat K50S-K51G encoding lentivirus, measured by Q-PCR.

Tat interacts with TAR through an arginine-rich domain located between amino acids 49 and 57. The association of Tat with human RNA could therefore occur through direct interaction through this RNA binding domain. To test this, we mutated lysines at positions 50 and 51 (K50S-K51G), which abrogates RNA binding activity [55]. Expression of this mutant in a LTR-reporter cell line showed that the K50S-K51G mutation reduced Tat activity by around 50%, verifying that the mutation compromised Tat function (Figure 4A). As we were unable to generate stable lines expressing Tat K50S-K51G, we instead expressed wild-type and K50S-K51G Tat in the T cell line CEM using a lentiviral vector. Flow cytometry indicated that the two viruses infected similar numbers of cells and western blotting for HA-Tat showed that the mutant form was expressed at higher rather than lower, levels than the WT protein (Figure 4B). Cell fractionation into nuclear and cytoplasmic components indicated that Tat K50S-K51G could still enter the nucleus, albeit at a slightly lower level (Figure 4C). HA-Tat was then precipitated from the cells as before and the associated RNA purified and quantified. We found that the K50S-K51G mutation reduced immunoprecipitation of RNA by 85%, down to the level observed with control non-specific antibody (Figure 4D). Although the use of transient transduction limited the number of cells available for analysis, we found that enrichment of the specific Tat-bound RNAs FADD and TNFRSF8 by HA IP was also abrogated by the K50S-K51G mutation (Figure 4E). These data demonstrate that the interaction of Tat with cellular RNA requires an intact RNA binding domain.

### Enrichment of a stem-loop structure within Tat-bound mRNAs

**Figure 5:**
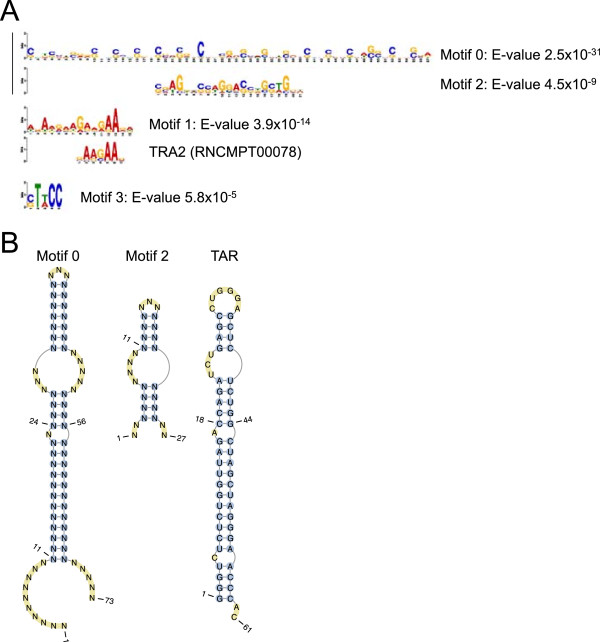
**Sequence motifs and stem-loop structures enriched in Tat-bound RNAs. A**. Sequence motifs significantly enriched within the set of Tat-bound mRNAs generated by MEME-ChIP. The numbering of the motifs (0 to 3) is ordered by their significance, with the E-values shown to the right. The motifs are represented as logos with the height of the letter representing the probability of the base at that position multiplied by the total information content of the position. Motifs 0 and 2 were determined by MEME-ChIP to be statistically similar and are aligned. Motif 1 was judged by MEME-ChIP to be similar to that of the splicing factor TRA2 (aligned). **B**. The consensus structure of all sequences that match motifs 0 and 2, determined by LocARNA. The structure of TAR (from [30]) is shown for comparison.

We next considered whether a sequence or structural motif could be shared between the set of Tat-bound RNAs, potentially explaining their shared precipitation by Tat RNA IP. We first sought to identify significantly enriched sequence motifs using the tool MEME-ChIP [56]. Analysing the set of RNAs bound in both HA and FLAG-Tat IPs, we identified 4 motifs that were significantly enriched (Figure 5A). Motif 2 was judged by MEME to be significantly similar to Motif 0 and thus they were grouped together. Comparison to motifs known to be recognised by RNA binding proteins [57] within MEME-ChIP failed to match motifs 0, 2 and 3 but motif 1 was found to be similar (p = 0.00017) to the motif identified for TRA2 [57], which regulates pre-mRNA splicing. MAST [58] shows that the motifs are often present at multiple copies within the bound RNAs and are distributed across the RNA molecules, rather than enriched towards the 5’ or 3’ ends (Additional file 3). Motif 0 is a long sequence with a striking periodic enrichment for C and G nucleotides. We considered that this sequence could form a stem-loop structure. We used FIMO [59] to identify the sequences within the set of Tat-bound mRNAs that matched Motif 0 and the related Motif 2 and used LocARNA [60] to discover whether these could be aligned and folded into common structures. The algorithm found that the sequences shared stem-loop structures (Figure 5B), which share similarities with TAR, including a bulge that in TAR (at position 23) is necessary for Tat binding [29,30]. Thus, the structure shared between Tat-bound cellular RNAs may play a role in the interaction with Tat.

### Human RNAs bound by Tat increase in abundance during HIV infection of primary CD4+ T cells

**Figure 6:**
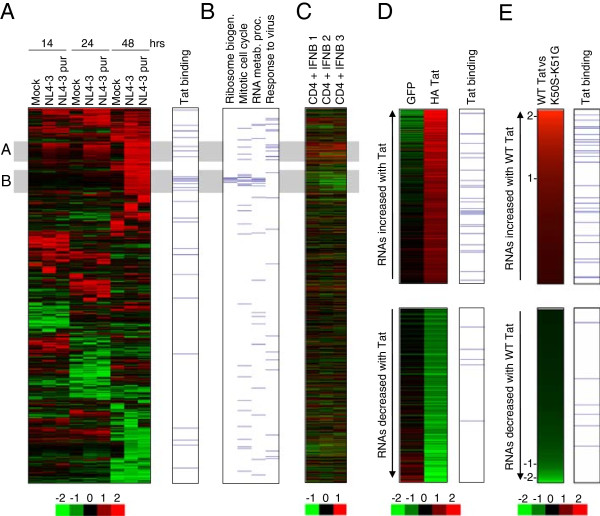
**RNAs associated with Tat show increases in transcript abundance. A**. Clustered heat-map showing changes in RNA abundance in primary CD4+ T cells after mock infection or infection with purified or unpurified HIV (NL3-4) at 14, 24 and 48 hours, relative to uninfected cells at the same time point. Shades of red indicate increases in RNA abundance and shades of green indicate decreases in RNA abundance, according to the scale below (log2 ratio). RNAs associated with Tat are marked by blue lines at the right hand side. **B**. Genes with functions in selected Gene Ontology categories are marked with a blue line. Data are aligned with A. **C**. Changes in RNA abundance in primary CD4+ T cells after addition of interferon-beta (400 U/ml) for 4 hours compared to untreated cells. Data for 3 replicate experiments are shown aligned with A. **D**. RNAs that increase in abundance (top) and decrease in abundance (bottom) by at least 1.5-fold in CEM cells stably expressing HA-Tat compared to parental CEM cells and CEM cells stably expressing GFP. The relative abundance of each RNA is represented by colour, according to the scale below (log2 ratio). RNAs associated with Tat are marked by blue lines at the right hand side. **E**. RNAs that increase in abundance (top) and decrease in abundance (bottom) in cells expressing WT HA-Tat compared to cells expressing the RNA binding mutant HA-Tat K50S-K51G. The 1000 RNAs with highest and lowest relative expression in WT Tat cells compared to K50S-K51G cells are shown. The relative abundance of each RNA (log2(WT-Tat vs Tat K50S-K51G) is represented by colour, with log2 ratios marked on the left. RNAs associated with Tat are marked by blue lines at the right hand side.

The interaction of Tat with human RNA may have functional consequences. Thus, we next asked whether RNAs bound by Tat show changes in abundance during HIV infection of primary CD4+ T cells. CD4+ T cells were purified from PBMC, activated with anti-CD3 and anti-CD28 antibodies, expanded with IL2 and then infected with both non-purified and purified stocks of HIV-1 (NL4-3) at a dose of 10 infectious units per cell. RNA was collected from infected and mock-infected cells at 14, 24 and 48 hours and changes in RNA abundance compared to uninfected cells measured using microarrays. Clustering RNAs by their expression patterns separates RNAs that exhibit increases in abundance during HIV infection from those that exhibit decreases in abundance (Figure 6A, Additional file 4). We observed two main clusters of RNAs that showed increases in transcript abundance after HIV infection, one from 14 hours and a second from 48 hours (labelled A and B in Figure 6A). Alignment of our Tat RNA binding data with the gene expression profiling data revealed that Tat was predominantly associated with RNAs that increased in abundance during infection (p = 0.003, hypergeometric) and was particularly associated with the 48 hour cluster (B in Figure 6A). Tat-associated RNAs that increased in abundance included TNFRSF8, MAPK12, METRNL, OAS3, BATF3, PLCG2, LTB, RASGRP2, JUN and PTGER1. Increased expression of a number of these genes has previously been implicated in HIV pathology [35-39].

We sought to characterise the two clusters of upregulated RNAs further to understand the significance of the association of Tat primarily with the second, later cluster. Gene Ontology revealed that genes in the first cluster upregulated by 14 hours have functions in the response to virus (5-fold enriched, p = 0.002), with the categories immune response (2.2-fold enriched, p = 0.0014) and regulation of programmed cell death (2.9-fold, p = 2.7×10^-5^) also enriched (Figure 6B and Additional file 5). Comparison with expression data from T cells exposed to interferon β showed that this cluster corresponds to the classical cellular interferon response to virus infection (Figure 6C).

In contrast, genes in the second cluster more strongly associated with Tat are instead enriched for RNAs encoding proteins with roles in ribosome biogenesis (19-fold enrichment, p = 6×10^-9^), RNA metabolic process (8-fold enrichment, p = 1.2×10^-4^) and mitotic cell cycle (6-fold enrichment, p = 1.4×10^-5^) (Figure 6B and Additional file 5). Contrary to their upregulation by HIV, these genes are instead downregulated by interferon (Figure 6C) and thus upregulation would appear to be specific for HIV infection. We considered that upregulation of these genes normally repressed by interferon might reflect an ability of Tat to counteract the normal interferon-stimulated response. However, gene expression changes induced by interferon in Tat-expressing CEM cells were similar to those in the parental cell line (Additional file 6). We conclude that Tat interacts with a specific set of cellular RNAs that are upregulated during HIV infection.

### Interaction with HIV Tat is associated with an increase in RNA abundance

The upregulation of Tat-associated genes during HIV infection suggests that this increase in RNA abundance occurs due to Tat. To test this, we examined the abundance of mRNAs in CEM cells stably expressing Tat compared with the parental CEM cell line and a CEM line stably expressing GFP, selected in parallel. We found that although the majority of Tat-associated mRNAs did not show changes in abundance in cells expressing Tat, significantly more RNAs exhibited an increase in abundance than expected by chance (n = 23, p < 0.001, Hypergeometric; Figure 6D, Additional file 7). RNAs bound by Tat that also increased upon expression of Tat included TNFRSF8, ISG20, MAPK12, METRNL and DNAJB2, all of which are also upregulated during HIV infection (Additional file 4, [35-39]). In contrast, there was no significant relationship between Tat binding and decreases in transcript abundance (n = 5, p = 0.95, Figure 6D). Furthermore, there was no relationship between the RNAs detected in control IPs and changes in RNA abundance in Tat-expressing cells (p = 0.178).We next considered whether Tat RNA binding activity was necessary for increases in RNA abundance. We constructed two stable cell lines, in which expression of either WT Tat or the Tat RNA binding mutant K50S-K51G could be induced by treatment with Doxycycline and measured RNA abundance using gene expression microarrays (Figure 6E). We found that cells expressing WT Tat exhibited changes in RNA abundance compared with cells expressing Tat K50S-K51G, with a greater number of genes showing increased expression in the WT Tat cells (Figure 6E). Furthermore, changes in RNA abundance were correlated with detection of Tat binding to the RNA, with Tat being bound more frequently to the set of RNAs exhibiting increased abundance in WT Tat cells compared to RNAs that exhibited decreased abundance. We conclude that Tat RNA binding is required for Tat to increase RNA abundance.

## Discussion

Although many interactions between HIV and host cell proteins have been described, whether the virus interacts with cellular RNAs was unknown. We have discovered that HIV Tat interacts with a set of cellular mRNAs in human T cells. These interactions are specific because they can be detected using two different epitope tags, they are not observed in control IPs, and the interactions can be verified by Q-PCR. The interaction of Tat with cellular RNA depends on lysine residues within the RNA binding domain known to be important for interaction with TAR. We could not detect association of Tat with miRNAs or with protein components of RISC. The cellular mRNAs bound by Tat are associated with specific molecular functions and share predicted stem-loop elements. Tat expression and HIV infection frequently results in an increase in the abundance of Tat-bound RNAs.

Tat has previously been shown to interact with three human RNAs by gel-shift assays *in vitro*[31-33] but such experiments lack specificity and whether these few interactions are representative of those occurring in cells was not clear. The system we have developed, in which RNA is purified from immunoprecipitated Tat tagged with independent epitopes and expressed from its own promoter, allowed us to detect the specific interactions between physiological levels of Tat and cellular RNA as they occur in cells. Indeed, although these experiments show that Tat interacts with a select set of human RNAs in cells, we could not detect an interaction between Tat and the RNAs with which it has previously been reported to interact *in vitro*[31-33].

Tat binding to TAR tolerates mutations to the stem and loop and, although the bulge structure at position +23 is required, Tat recognises a number of variants [29,30]. Tat also binds related structures predicted to form within the IL6 and TLR4 mRNAs [31,32]. Analysis of motifs significantly enriched in the set of Tat-bound RNAs revealed a striking palindromic GC rich motif that is predicted to fold into stem-loop structures similar to TAR (Figure 5). We found that these motifs were located throughout Tat target RNAs, rather than being located in at the 5’ end as for TAR. Therefore, if Tat does recognise such RNA elements, they are unlikely to function in the same way as TAR in the activation of transcriptional elongation. Identification of Tat-RNA UV-crosslink sites at high-resolution using sequencing-based methods such as iClip or PAR-Clip [61] will confirm whether Tat is binding to these elements and whether this occurs in live cells. Their importance for Tat binding could then be tested through the expression of RNAs in which such structures are disrupted by mutation.

Tat has previously been shown to cause changes to human gene expression and some of these changes have been linked to HIV pathology [35-39]. How Tat mediates such changes in RNA abundance is unclear. In addition to being an RNA binding protein, Tat has also been reported to interact with a great many proteins, from transcription factors to cell surface receptors [40-49], and so Tat-mediated changes in gene expression could theoretically be due to these interactions rather than Tat RNA binding function. Arguing against this, we have identified a global association between Tat binding to cellular mRNAs and an increase in RNA abundance. Firstly, a number of RNAs previously shown to be upregulated by HIV and by Tat are also bound by Tat, including ISG20, JUN, DNAJB2, ISG20, MAPK12, METRNL, TNFRSF8 and OAS3. Secondly, Tat is associated with a cluster of RNAs upregulated during HIV infection. Thirdly, we detect Tat binding at a significant number of the RNAs that increase in abundance in Tat-expressing cells. Finally, mutations that block Tat RNA binding activity counteract these increases in RNA abundance. Thus, the association of Tat with specific mRNAs provides a direct link between Tat and changes in RNA abundance during HIV infection that is parsimonious with its core RNA binding function. Tat binding to host RNA may act to recruit or displace a cellular factor or otherwise alter mRNA transcription, processing, export or stability. Further experiments will be required to identify the mechanism through which Tat RNA binding leads to changes in cellular mRNA abundance.

Early studies of Tat function considered the hypothesis that Tat might counteract the host interferon response [62] and there is some evidence Tat can regulate interferon target genes. Treatment of cells with recombinant Tat has been suggested to suppress IFNγ signaling through upregulation of SOCS2 [63] and Tat has been reported to interact with PKR, modulating its function [64]. Expression of Tat in dendritic cells leads to induction of interferon-inducible genes encoding T cell chemo-attractants, luring these target cells to sites of infection [36]. We thus examined whether Tat bound to interferon-regulated RNAs, and whether the presence of Tat altered the transcriptional response to interferon-β in our T cell line model. However, we did not find evidence that Tat RNA binding acts to modulate the transcriptional response to interferon (Additional file 6).

Although we could detect interaction of Tat with TAR and cellular mRNAs and detect interaction of Ago2 with miRNAs, we could not detect interaction of Tat with cellular miRNAs. This suggests that Tat either does not interact with miRNAs or that the interaction occurs at a low level beyond our detection. Consistent with the lack of detected Tat-miRNA interactions, we also failed to identify an interaction between Tat and endogenous Dicer, Ago1 or Ago2. Tat has previously been reported to directly interact with exogenously expressed Myc-tagged Dicer [17], but interaction with the endogenous protein has not been described. Whether or not Tat inhibits RNA silencing is also unclear; some studies report that Tat can repress small RNA generation [16-19], but others have been unable to confirm this [20,21]. Our data indicate that if Tat does act to inhibit PTGS, it is unlikely to do so through an association with cellular miRNAs.

Our discovery that Tat targets a specific set of cellular RNAs presents a new interface through which HIV can interact with its host. In addition to Tat, HIV encodes two other RNA binding proteins, Rev and nucleocapsid, which may also interact with cellular RNAs in addition to their viral targets. A number of other viruses encode RNA binding proteins that may also target specific cellular RNAs. For example, Hepatitis C virus (HCV) NS5a binds to the 3’-ends of HCV plus and minus strand RNAs and functions in genomic RNA replication but has also been implicated in the regulation of host genes, including upregulation of *IL8* and *LTB*[65,66]. Influenza NS1 interacts with viral mRNAs encoding M1 and NS1 but can also interact with polyA RNA, dsRNA and human U6 and U6atac snRNAs, functioning to inhibit pre-mRNA splicing, 3’-end processing and mRNA export (reviewed in [67]). Epstein-Barr virus (EBV) SM protein binds to unspliced viral RNA to allow export from the nucleus but also interacts with cellular STAT1 mRNA, inducing a splicing reaction that leads to production of the STATβ isoform that can act as a dominant-negative suppressor of STAT1α [68]. The methods we have presented here could be used to determine whether these viral factors and others also interact with a specific set of host RNAs.

The mRNAs bound by Tat encode a set of proteins enriched for specific functions including nucleotide binding and tRNA metabolic processes. It is unclear why HIV Tat should target these RNAs in particular but similar functional classes of gene are also upregulated upon HIV infection (Figure 6B and Additional file 5). This may reflect the virus’s need to create an environment in which large amounts of viral genomic RNA and proteins can be efficiently synthesised. As such, the interaction of Tat with specific cellular RNAs may have been selected during evolution because it aids HIV replication. Alternatively, interactions between Tat and cellular RNAs may instead represent off-target effects of Tat’s TAR binding function that offers no selective advantage to the virus. Either way, the interaction of Tat with cellular RNA likely has consequences for the host cell. Further studies will be required to determine whether the association of Tat with specific cellular RNAs promotes virus replication or pathogenesis.

## Conclusions

We have discovered that HIV Tat interacts with a specific set of cellular mRNAs. These interactions are detected regardless of the antibody used to purify Tat and occur when Tat is expressed at physiological levels. Tat does not interact with cellular miRNAs or with RISC components. Tat binding is linked to increases in mRNA abundance, both in Tat-expressing cells and during HIV infection of primary CD4+ T cells. The interaction of Tat with human RNA reveals a new interaction between HIV and its host. Given Tat’s pleiotropic effects on the host cell, this has implications for the pathology of this important viral agent. The interaction between viral proteins and host RNA may in future prove to be a common feature of virus infection.

## Methods

### Cells lines and plasmids

The promoter in pIRESneo3 was replaced by the LTR from HIV SF2 and HA or FLAG-tagged 101-amino acid Tat (from SF2) inserted downstream (before IRES-G418R). These constructs were transfected into CEM cells and stable lines selected. For RNA IP experiments measuring the role of Tat RNA binding function, HA-Tat was cloned into the pHR-SIN-CSGW-EGFP lentiviral expression construct [69] in which the EGFP cassette was replaced by IRES-emGFP and K50S-K51G mutations made by site-directed mutagenesis (Stratagene). The vectors were transfected into 293T cells together with the packaging plasmids pMDG and p8.91 and virus harvested, concentrated and titred on CEM cells. CEM cells were infected with the viruses with an MOI of 10. To quantify the expression in CEM cells relative to HIV infected cells, the HA tag was also inserted into the 5’ end of Tat in the p9B18 clone of HIV strain SF2. To compare changes in RNA abundance associated with Tat RNA binding activity, HA-Tat and HA-Tat K50S-K51G were cloned into pTRE2Hyg (Clontech), transfected into Jurkat Tet-On cells and stable lines selected.

### HIV infection of primary CD4+ T cells

pNL4-3 encoding the NL4-3 strain of HIV was transfected into 293T cells and virus purified by pelleting through sucrose and titred on GHOST cells. CD4+ T cells were purified from PBMC (Buffy coat) by negative selection (Miltenyi), activated with anti-CD3 and anti-CD28 (1ug/ml) for 60 hours and then expanded in IL2 (5 ng/ml) for 48 hours. Cells were then split and infected with unpurified NL4-3 (contained within 293T cell supernatant), NL4-3 purified on a sucrose cushion (both with a dose of 10 infectious units per cell) or mock-infected by addition of 293-conditioned media. Virus was spinoculated at 1000 g for 1 hour and infection was allowed to proceed for 1 hour at 37°C. Virus was then washed off the cells and the cells incubated in fresh media. Cells were harvested in TRIzol (Invitrogen) at 14, 24 and 48 hrs after infection. We used purified HIV virus in addition to non-purified virus so we could filter out expression changes in the recipient T-cells potentially induced by cytokines secreted by the producer 293T cells. In fact, we didn’t observe any significant differences in the response to the two viral stocks and so both datasets were used in the analysis. After activation, non-infected primary CD4+ T cells were also treated by addition of interferon-beta (400 U/ml) for 4 hours.

### RNA IP

CEM cells expressing HA or FLAG-tagged Tat were lysed (10 mM Hepes, 100 mM KCl, 5 mM MgCl2, 0.5% NP-40, 1 mM DTT, Complete protease inhibitor (Roche) and RNaseOUT (Invitrogen)) and the nuclei sheared through a 27-guage needle and by freeze-thawing, as described [70]. The lysates were then DNase treated (Ambion) on ice for 30 mins, diluted in NT buffer (50 mM Tris pH7.5, 150 mM NaCl, 1 mM MgCl_2_, 0.05% NP-40, supplemented as before) and insoluble material removed by centrifugation. Lysates were precleared with protein-G beads (Dynal) and Tat IP performed with anti-HA (3F10; Roche) or anti-FLAG (M2; Sigma) antibody. Beads were washed 4 times with NT2 and twice with NT2 containing 1 M urea. Total RNA was purified from beads and input cell lysate with Trizol LS (Invitrogen), DNaseI treated and the integrity verified using an Agilent Bioanalyzer.

CEM cells crosslinked with 1% formaldehyde were lysed in ChIP lysis buffer [70] in the presence of RNaseOUT, sonicated at 9 W for 3 mins (20s pulses) and insoluble material removed by centrifugation. The lysate was precleared and then incubated with 3F10 or control Rat IgG (Santa Cruz) for 16 hours. The beads were washed as described [70] and the crosslinks reversed by incubation at 65°C for 1 hour. RNA was then purified with TRIzol LS and DNaseI treated.

### Microarray analysis of RNA IP samples

200 ng of IP or input RNA were labelled using Agilent’s QuickAmp labelling kit and hybridized to DNA microarrays (Agilent). Initial HA-Tat and FLAG-Tat IPs were hybridized to a custom microarray containing multiple probes to all RefSeq mRNAs (includes standard Agilent probes) [70]. For these custom arrays, RefSeq mRNAs with at least one probe with an IP/input ratio above 2-fold and p-value < 0.001 and at least one other with an IP/input ratio above 1.5-fold and p-value < 0.001 were judged to be enriched by the IP. Subsequent dye-swap and control IPs were analysed using catalogue Agilent 44K human gene expression arrays (AMADID 014850). For these arrays, the ratio and p-values were averaged for RefSeq mRNAs with multiple probes and then those mRNAs with an IP/input ratio above 2 and p-value < 0.001 were judged to be enriched by the IP. Log2 IP/input ratios were filtered to remove those with insignificant p-values and the remaining data clustered using average-linkage hierarchical clustering. A total of 3 Tat RNA IP microarray experiments were performed.

### miRNA arrays

100 μg of input and IP RNA was labelled and hybridized to Agilent human miRNA arrays following the standard protocol. The log2 ratio of the miRNA signal to the geometric mean signal across all samples was calculated and samples and miRNAs clustered by average-linkage hierarchical clustering.

### Gene expression analysis

Total RNA was purified from cells using Trizol, DNase treated (Ambion) and integrity confirmed. The HIV samples were hybridized together with reference RNA (Stratagene). The expression ratio between infected cells and untreated cells at the same timepoint was calculated, data for genes with multiple probes averaged and probes filtered for those exhibiting at least 1 absolute log2 expression ratio above 1. The CEM cell line material was hybridized together with RNA from the parental CEM line. Genes were judged to be differentially expressed if HA-Tat/CEM and HA-Tat/GFP were both above 1.5 or both below 0.66. Tat expression was induced in Jurkat cells with 1 μg/ml Doxycycline for 48 hours and hybridised with RNA from untreated cells. Gene Ontology analysis was performed using DAVID [71].

### Q-PCR

cDNA was synthesized with Superscript II (Invitrogen) and random primers. Primer and probes were from Applied Biosystems; FADD (Hs00356603_g1), ISG20 (Hs00158122_m1), TNFRSF8 (Hs01114493_m1) and HPRT1 (4333768) or custom synthesized; TAR (F:GCTAACTAGGGAACCCACTGCTT, R: CAACAGACGGGCACACACTACT, Probe: AGCCTCAATAAAGCTTGCCTTGAGTGCTTC), GAPDH (F: GGCTGAGAACGGGAAGCTT, R:AGGGATCTCGCTCCTGGAA, Probe: TCATCAATGGAAATCCCATCACCA), 7SK RNA (F:AGAACGTAGGGTAGTCAAGC, R:AGAAAGGCAGACTGCCACAT).

### Co-immunoprecipitation, cell fractionation and immunoblotting

Cells were washed in PBS and lysed with 40 mM Tris pH 7.5, 150 mM NaCl, 10% glycerol, 0.3% NP-40 containing Complete protease inhibitors (Roche) for 20 min, and insoluble material removed by centrifugation. Supernatants were incubated with anti-HA (3F10) antibody bound to magnetic beads at 4°C for 16 hrs. Beads were then washed successively in IP buffer, IP buffer containing 300 mM NaCl, IP buffer containing 500 mM NaCl 0.1% NP-40, and then with 20 mM Tris–HCl pH 8.0, 150 mM NaCl, 10% glycerol. Lysates were separated by SDS-PAGE and proteins detected with the following antibodies: HA-Tat (Roche 3F10), FLAG (Sigma M2), TBP (Abcam ab818), Tubulin (Sigma T4026), Dicer (Abcam ab14601), Ago1 (Millipore 07–599) and Ago2 (Millipore 07–590). CEM cells were fractionated in nuclear and cytoplasmic fractions as described [72].

### Motif discovery

The set of 317 enriched mRNAs in both HA and Tat RNA IP were filtered to contain one transcript per gene (the longest transcript was selected) and run through MEME-ChIP [56] with the default options, except for scan given strand only, any number of repetitions per sequence, maximum width of 60, and compare to the motif database of Ray et al. [57]. The distribution of motifs across the RNAs was determined using MAST [59], with an E-value of 1 and the options: use individual sequence composition, ENSEMBL homo sapiens database, scale motif display threshold and no treatment of reverse complement strands. To identify sequences within the bound RNAs that match the discovered motifs, we used FIMO [60] with the ENSEMBL homo sapiens database and scan given strand only option. We then selected the 143 sequences matching with motif 0 with a p-value of <10^-9^, or the 58 sequences that matched motif 2 with p < 10^-6^, and aligned these with LocARNA, using the global option [61]. The consensus structures identified by LocARNA were then visualised with Pseudoviewer [73].

### Availability of supporting data

The data sets supporting the results of this article are available in the ArrayExpress repository, accession number E-MEXP-3199.

## Competing interests

The authors declare that they have no competing interests.

## Authors’ contributions

RGJ conceived the study. RDB, AP, CP, EC, JR and RGJ performed experiments. AP, PK and RGJ analysed and interpreted data. RGJ wrote the manuscript with input from all authors. All authors read and approved the final manuscript.

## Supplementary Material

Additional file 1 **Human RefSeq mRNAs associated with HIV Tat in CEM T cells.** Transcript IDs, Entrezgene ID, Gene name and Description of mRNAs bound by HIV Tat in replicate experiments. Click here for file

Additional file 2 **Tat bound RNAs of the KEGG aminoacyl tRNA biosynthesis pathway.** Schematic of the aminoacyl tRNA biosynthesis pathway defined by the Kyoto Encyclopedia of Genes and Genomes (KEGG). Proteins encoded by Tat-bound RNAs (from the expanded set of 2000 RNAs) are highlighted in green. Proteins are named by their IUBMB enzyme nomenclature. Click here for file

Additional file 3 **Location of sequence motifs enriched in Tat-bound mRNAs.** MAST was used to identify the position of sequences matching each of the motifs (Figure 5). Each of the mRNAs shown has an E-value less than 0.001. The motif matches shown have a position p-value less than 0.01. Motif 3 was removed by MAST because it had a similarity greater than 0.60 with another motif (Motifs 0 and 2). Click here for file

Additional file 4 **HIV infection gene expression data for Figure** 6**A.** Transcript IDs, Entrezgene ID, Gene name, Description and log2 gene expression ratios relative to untreated cells at the same timepoint. Data for genes with multiple probes are averaged and probes filtered for those exhibiting at least 1 absolute log2 expression ratio above 1. Click here for file

Additional file 5 **Gene Ontology biological process categories enriched in the sets of genes upregulated during HIV infection.** A. As Figure 6B, except showing genes with functions in an expanded set of Gene Ontology categories. B. P-values of Gene Ontology biological process categories enriched (p < 0.001) in the A and B clusters marked in A. Enrichment of each functional category is specific to only one of the two clusters. Click here for file

Additional file 6 **Tat RNA binding is not associated with changes in the abundance of interferon-inducible RNAs.** Scatter plot of the changes in RNA abundance in CEM cells treated with IFNβ (400U/ml for 4 hours) versus change in RNA abundance in CEM-HA-Tat cells treated with IFNβ. RNAs associated with Tat by native RNA IP are indicated in red. RNAs bound by Tat are not differentially regulated by IFNβ compared with other RNAs and also do not show significant differences in their response to IFNβ in the presence of Tat. Click here for file

Additional file 7 **CEM HA-Tat gene expression data in Figure** 6**D.** Transcript IDs, EntrezGene ID, Gene name, Description and log2 gene expression ratios relative to the parental CEM cell line and the CEM-GFP cell line. RNAs associated with HIV Tat (by RNA IP) are indicated. Click here for file
