# Supplementary figures and images for: Human immunodeficiency virus Tat associates with a specific set of cellular RNAs
Source: Retrovirology. 2014 Jul 3;11:53. doi: 10.1186/1742-4690-11-53 (PMC4086691; doi:10.1186/1742-4690-11-53)

# AMINOACYL-tRNA BIOSYNTHESIS

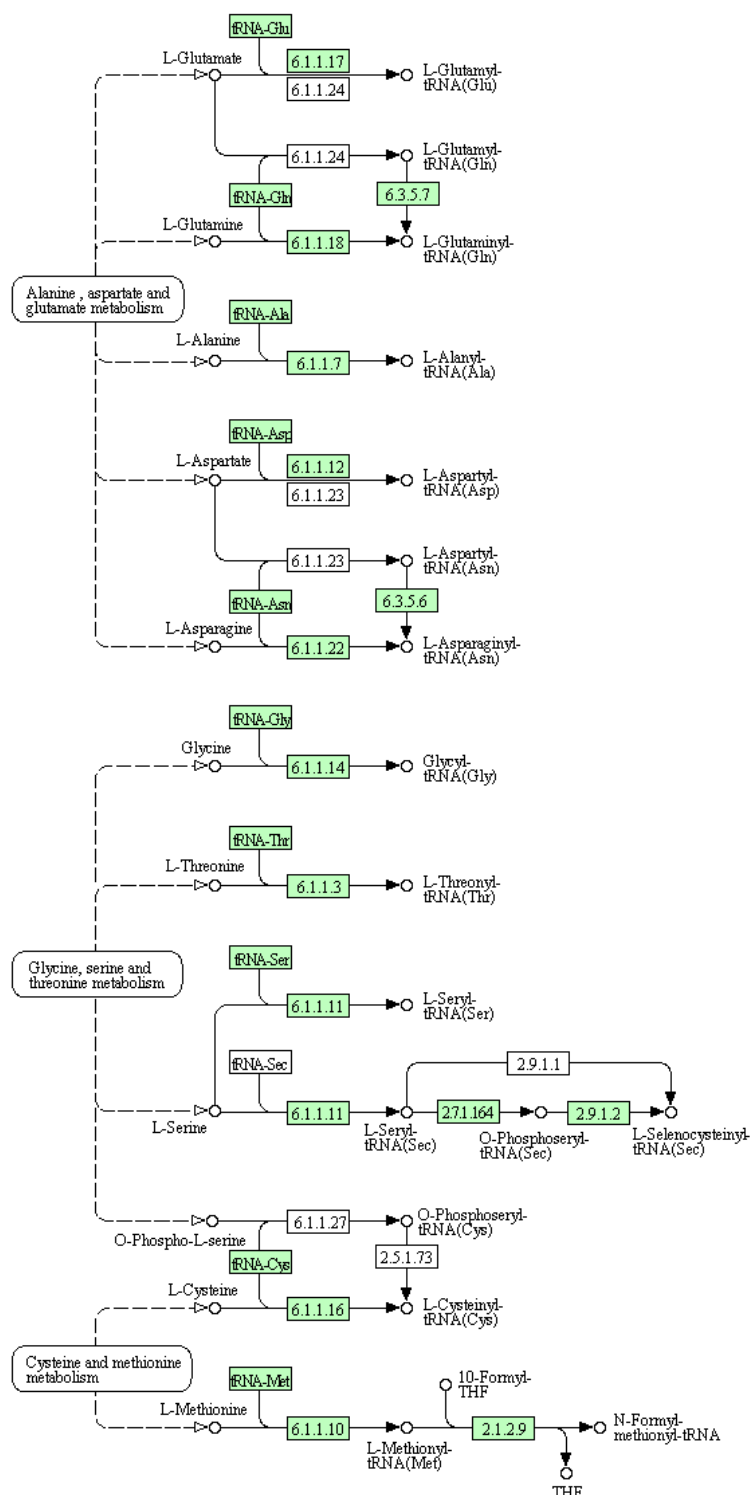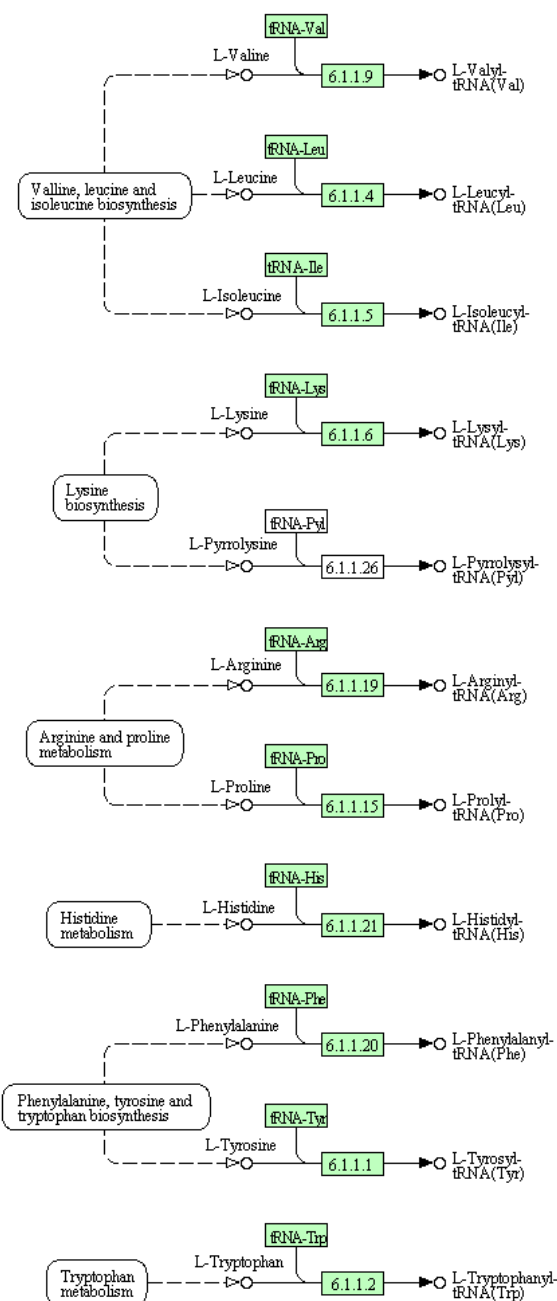

Supplement: Additional file 2 — Tat bound RNAs of the KEGG aminoacyl tRNA biosynthesis pathway. Schematic of the aminoacyl tRNA biosynthesis pathway defined by the Kyoto Encyclopedia of Genes and Genomes (KEGG). Proteins encoded by Tat-bound RNAs (from the expanded set of 2000 RNAs) are highlighted in green. Proteins are named by their IUBMB enzyme nomenclature. [file 1742-4690-11-53-S2.pdf]

A

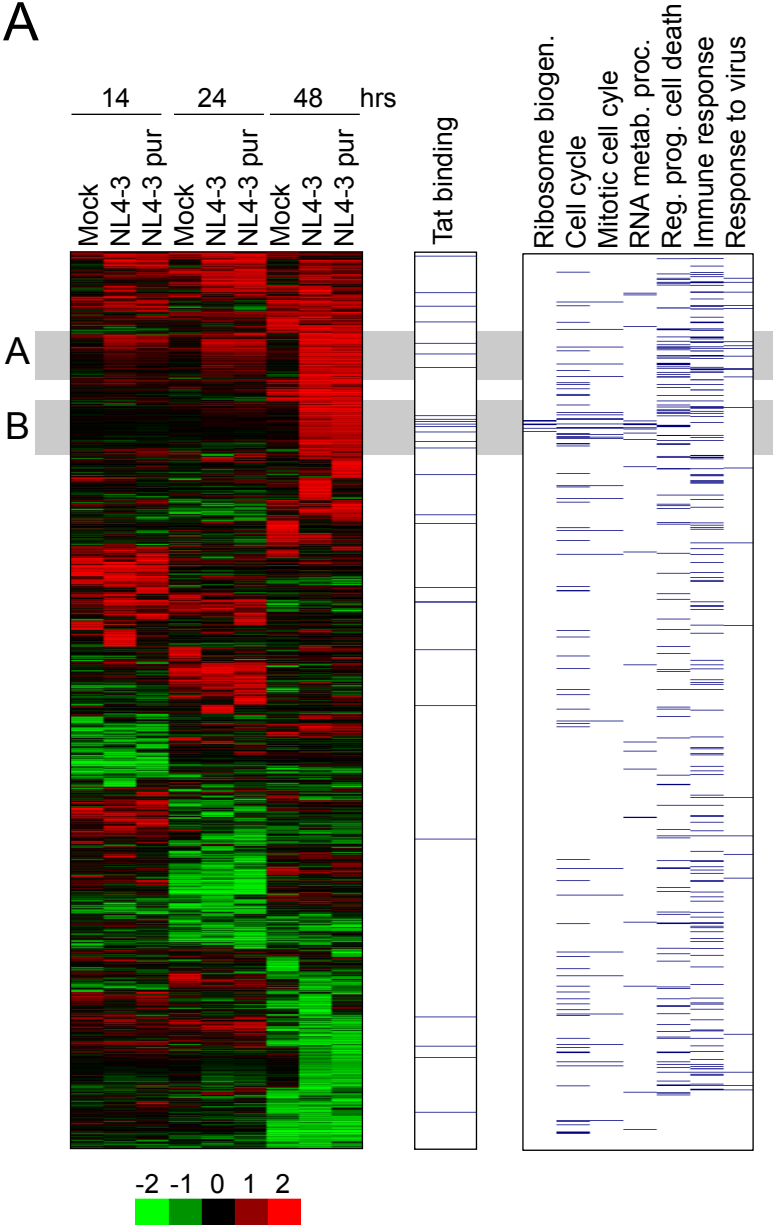

B

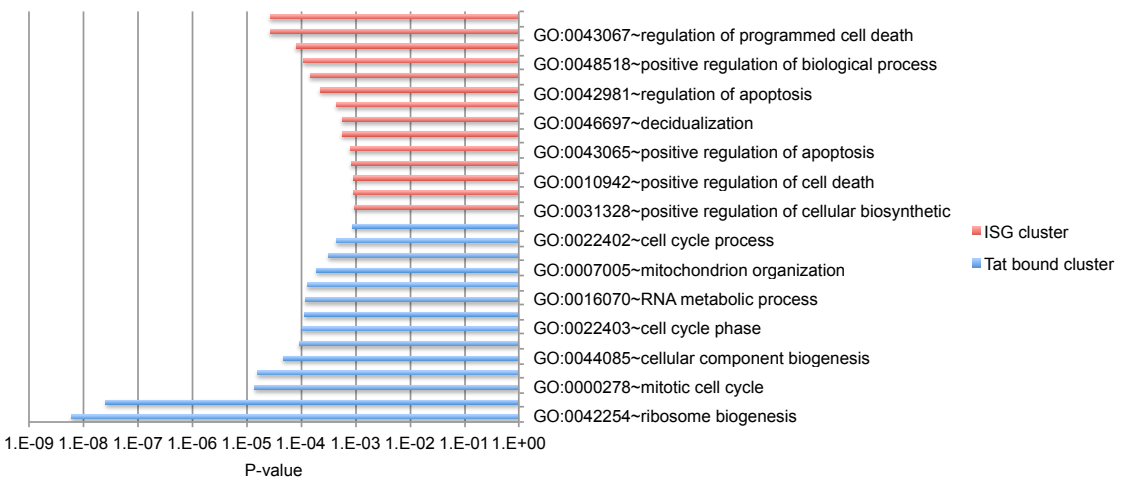

Supplement: Additional file 5 — Gene Ontology biological process categories enriched in the sets of genes upregulated during HIV infection. A. As Figure 6B, except showing genes with functions in an expanded set of Gene Ontology categories. B. P-values of Gene Ontology biological process categories enriched (p < 0.001) in the A and B clusters marked in A. Enrichment of each functional category is specific to only one of the two clusters. [file 1742-4690-11-53-S5.pdf]

$\log_2(\text{CEM-Tat} + \text{IFNB} / \text{CEM-Tat})$

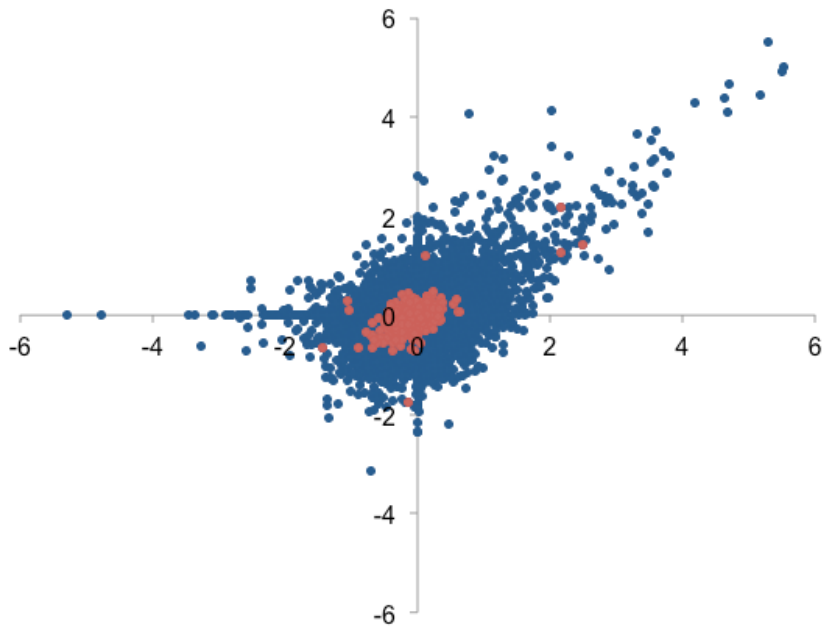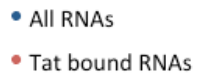

$\log_2(\text{CEM} + \text{IFNB} / \text{CEM})$

Supplement: Additional file 6 — Tat RNA binding is not associated with changes in the abundance of interferon-inducible RNAs. Scatter plot of the changes in RNA abundance in CEM cells treated with IFNβ (400U/ml for 4 hours) versus change in RNA abundance in CEM-HA-Tat cells treated with IFNβ. RNAs associated with Tat by native RNA IP are indicated in red. RNAs bound by Tat are not differentially regulated by IFNβ compared with other RNAs and also do not show significant differences in their response to IFNβ in the presence of Tat. [file 1742-4690-11-53-S6.pdf]
