# Supplementary material for: Human immunodeficiency virus Tat associates with a specific set of cellular RNAs
Source: Retrovirology. 2014 Jul 3;11:53. doi: 10.1186/1742-4690-11-53 (PMC4086691; doi:10.1186/1742-4690-11-53)

Sequence	*E*-value
lcl|NM_203500.1_cdsid_NP_987096.1	1e-07
lcl|NM_014053.3_cdsid_NP_054772.1	4.1e-06
lcl|NM_014015.3_cdsid_NP_054734.2	4.5e-06
lcl|NM_002691.3_cdsid_NP_002682.2	4.5e-06
lcl|NM_001008697.1_cdsid_NP_001008697.1	1.4e-05
lcl|NM_006753.5_cdsid_NP_006744.2	1.8e-05
lcl|NM_014608.3_cdsid_NP_055423.1	1.9e-05
lcl|NM_013282.4_cdsid_NP_037414.3	2.3e-05
lcl|NM_001671.4_cdsid_NP_001662.1	3.4e-05
lcl|NM_000455.4_cdsid_NP_000446.1	3.6e-05
lcl|NM_006821.5_cdsid_NP_006812.3	3.7e-05
lcl|NM_014262.4_cdsid_NP_055077.2	4.8e-05
lcl|NM_014909.4_cdsid_NP_055724.1	5.5e-05
lcl|NM_021174.5_cdsid_NP_066997.3	5.7e-05
lcl|NM_001243.4_cdsid_NP_001234.3	6.2e-05
lcl|NM_020435.3_cdsid_NP_065168.2	8.4e-05
lcl|NM_005346.4_cdsid_NP_005337.2	0.00012
lcl|NM_144653.4_cdsid_NP_653254.1	0.00013
lcl|NM_025268.2_cdsid_NP_079544.1	0.00014
lcl|NM_025078.4_cdsid_NP_079354.2	0.00014
lcl|NM_006516.2_cdsid_NP_006507.2	0.00015
lcl|NM_002918.4_cdsid_NP_002909.4	0.00017
lcl|NM_015833.3_cdsid_NP_056648.1	0.00018
lcl|NM_003278.2_cdsid_NP_003269.2	0.0002
lcl|NM_003885.2_cdsid_NP_003876.1	0.00022
lcl|NM_002840.3_cdsid_NP_002831.2	0.00023
lcl|NM_006927.3_cdsid_NP_008858.1	0.00027
lcl|NM_024632.5_cdsid_NP_078908.1	0.0003
lcl|NM_005049.2_cdsid_NP_005040.2	0.00054
lcl|NM_001466.3_cdsid_NP_001457.1	0.00054
lcl|NM_032164.2_cdsid_NP_115540.2	0.00067
lcl|NM_015523.3_cdsid_NP_056338.2	0.00068
lcl|NM_003501.2_cdsid_NP_003492.2	0.00072
lcl|NM_003371.3_cdsid_NP_003362.2	0.00073
lcl|NM_022120.1_cdsid_NP_071403.1	0.0009
lcl|NM_018166.1_cdsid_NP_060636.1	0.00091
lcl|NM_006320.4_cdsid_NP_006311.2	0.00091
lcl|NM_000189.4_cdsid_NP_000180.2	0.00094
lcl|NM_001436.3_cdsid_NP_001427.2	0.00094
lcl|NM_005453.4_cdsid_NP_005444.4	0.00096
lcl|NM_015721.2_cdsid_NP_056536.2	0.00098
lcl|NM_002969.3_cdsid_NP_002960.2	0.00099

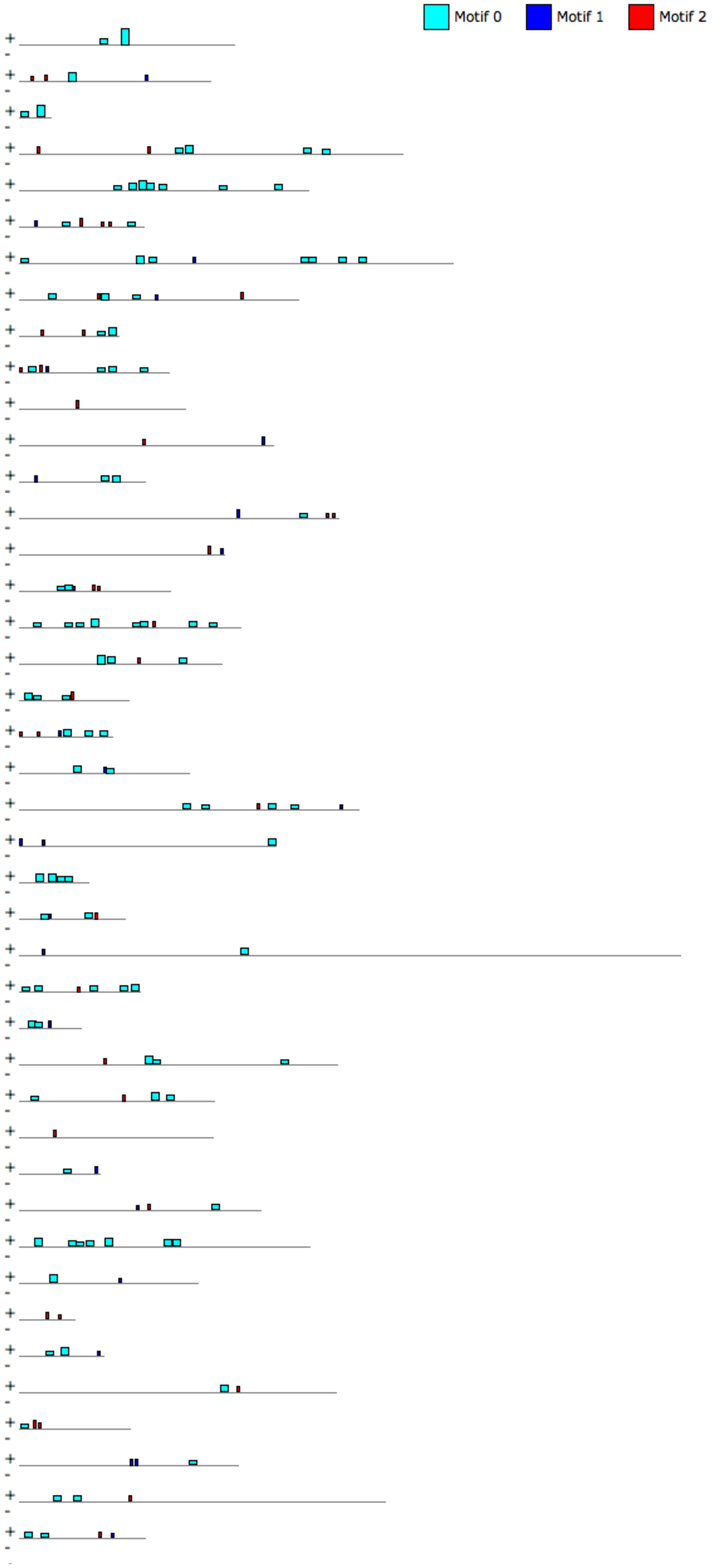

Supplement: Additional file 3 — Location of sequence motifs enriched in Tat-bound mRNAs. MAST was used to identify the position of sequences matching each of the motifs (Figure 5). Each of the mRNAs shown has an E-value less than 0.001. The motif matches shown have a position p-value less than 0.01. Motif 3 was removed by MAST because it had a similarity greater than 0.60 with another motif (Motifs 0 and 2). [file 1742-4690-11-53-S3.pdf]
